# Reanalysis of genomic data, how do we do it now and what if we automate it? A qualitative study

**DOI:** 10.1038/s41431-023-01532-4

**Published:** 2024-01-12

**Authors:** Zoe Fehlberg, Zornitza Stark, Stephanie Best

**Affiliations:** 1 Australian Genomics, Murdoch Children’s Research Institute, Melbourne, VIC Australia; 2 University of Melbourne, Melbourne, VIC Australia; 3 Victorian Clinical Genetics Services, Murdoch Children’s Research Institute, Melbourne, VIC Australia; 4 Department of Health Services Research, Peter MacCallum Cancer Centre, Melbourne, VIC Australia; 5 Victorian Comprehensive Cancer Centre, Melbourne, VIC Australia; 6 Department of Oncology, Sir Peter MacCallum Cancer Centre, University of Melbourne, Melbourne, VIC Australia

**Keywords:** Genetic testing, Genetic testing

## Abstract

Automating reanalysis of genomic data for undiagnosed rare disease patients presents a paradigm shift in how clinical genomics is delivered. We aimed to map the current manual and proposed automated approach to reanalysis and identify possible implementation strategies to address clinical and laboratory staff’s perceived challenges to automation. Fourteen semi-structured interviews guided by a simplified process map were conducted with clinical and laboratory staff across Australia. Individual process maps were integrated into an overview of the current process, noting variation in service delivery. Participants then mapped an automated approach and were invited to discuss perceived challenges and possible supports to automation. Responses were analysed using the Consolidated Framework for Implementation Research, linking to the Expert Recommendations for Implementing Change framework to identify theory-informed implementation strategies. Process mapping demonstrates how automation streamlines processes with eleven steps reduced to seven. Although participants welcomed automation, challenges were raised at six of the steps. Strategies to overcome challenges include embedding project champions, developing education materials, facilitating clinical innovation and quality monitoring tools, and altering reimbursement structures. Future work can build on these findings to develop context specific implementation strategies to guide translation of an automated approach to reanalysis to improve clinical care and patient outcomes.

## Introduction

Reanalysis is a process of re-examining unsolved patient’s existing genomic data in light of advancements in knowledge and analytic tools, thus maximising diagnostic yield in rare disease over time. With expanding use of genomic testing and diagnostic yield under 50%, [1] the ability to periodically reanalyse stored genomic data manually becomes less feasible. Automating reanalysis has been a highly anticipated solution [2]. Notwithstanding interest, translating automated reanalysis into clinical practice has proven difficult and presents a fundamental change in how clinical genomics is delivered. The challenge for healthcare systems is striking a balance between maximum clinical impact, minimising additional workloads, and securing acceptable financial reimbursement [3]. Other issues include, having bioinformatics and health informatics technology and systems in place to be able to process the increased volume of data, and the ethical, legal, and logistical considerations regarding consent and recontacting patients if new findings are found [4–6]. Laboratories and clinical services raise workforce scope and capacity with estimates that reanalysis requires a highly skilled workforce plus 20–40 h to re-evaluate data [7, 8] increasing clinicians’ workloads to assist in clinical interpretation of variants of interest [9] and provide counselling [10].

Clinical genomics, like many areas of healthcare operates in a complex setting, sensitive to a range of contextual factors [11]. It is often these factors that cause real-world implementation efforts failure or success [12]. Applying implementation science frameworks provides a systematic and theory-informed approach to identifying factors influencing implementation (or barriers) and selecting appropriate and context specific strategies to address implementation challenges. Two frameworks used in tandem for this purpose are, the Consolidated Framework for Implementation Research (CFIR) [13] and the Expert Recommendation for Implementing Change [14]. Whilst prospective use is less common [15] integrating context assessment and identification of barriers and facilitators using the CFIR throughout the implementation process can guide tailored implementation strategies. The ERIC, amongst other uses, contains a matrix that links CFIR factors to possible implementation strategies. For example, ‘Patient Needs and Resources’ corresponds with ‘Obtain and use patients/consumers and family feedback’. Once ERIC strategies are selected, they can be designed to suit the local context, implemented, and evaluated for success. Ascertaining intuitively devised or experienced-based ideas or examples from those working in the field may help to optimise the design and translation of prospective strategies [16].

In this study, we aimed to i) process map the current manual and proposed automated approach to reanalysis, and ii) identify potential theory-informed implementation strategies to address clinical and laboratory staffs’ perceived challenges to automation.

## Methods

### Study setting

As of 2020, the Australian Commonwealth Government commenced funding for two cycles of reanalysis limited to patients <15 years old and at least 18 months after initial testing [17]. Previously, the cost of reanalysis was covered either by clinical services, state government health departments, research projects, or on a user-pays basis. We conducted this study within an Australian research project investigating the development and evaluation of a national automated reanalysis programme. The study employed a hybrid 1 effectiveness-implementation study design [18] to allow for the dual collection and assessment of clinical and implementation outcomes. Through the project, upwards of 10,000 rare disease patients and relatives will have their genomic data reanalysed using a continuously updated pipeline, with the aim of informing future policy. As part of the study, a landscape analysis was conducted to understand current clinical and laboratory practice, and attitudes towards automation.

### Research design

This study used a deductive qualitative study design combining two implementation research methods, process mapping and semi-structured interviews. Process mapping provided a nuanced insight into process variation and what needs to happen differently [19] and interviews facilitated in-depth exploration of stakeholders’ perceptions of the processes. Our work was underpinned by a constructivist paradigm [20] where participants knowledge is experientially generated. For analysis, we applied the CFIR which consists of constructs housed within five domains and are contextualised for the study as follows. The Innovation, an automated reanalysis programme; The Outer Setting, the Australian Health Care system; The Inner Setting, the laboratory or health service; The Individual, the laboratory or clinical staff; and The Process, implementing an automated reanalysis programme.

### Participants and recruitment

We invited clinical and laboratory staff to participate in semi-structured interviews prior to the implementation of the automated reanalysis programme. A purposeful criterion-sampling and snowball strategy was used to identify information-rich participants [21] who had reanalysis experience from paediatric and adult rare-disease and cancer genetics settings. At the end of interviews, participants were asked to nominate individuals to speak with from settings not yet captured and they were invited if eligible. Participants were invited via email by members of the study team with two follow-up invitations sent as required.

### Data collection tools and procedure

A simplified process map outlining the patient journey from ‘unsolved patient’ to ‘patient informed about result’ was shared with participants prior to, and during the interview to guide discussion [22]. An accompanying interview schedule (Supplementary Material 1) moved through the map to cover the current process for reanalysis at the participant’s workplace. The activity was repeated enquiring about the automated process, and participants thoughts on implementation in routine care (e.g., what do you think will be the main challenges to automation? Is there anything that could support implementation? What is the feeling in your workplace towards automation?). Questions were asked reflexively to adapt to participants’ roles. Interviews were conducted between May – June 2022 by experienced qualitative researchers (ZF, SB) who for some participants they knew professionally. Interviews were allotted 60 min, held via videoconference at the participants’ convenience, audio-recorded, de-identified, and transcribed verbatim by the study team.

### Data analysis

**Fig. 1:**
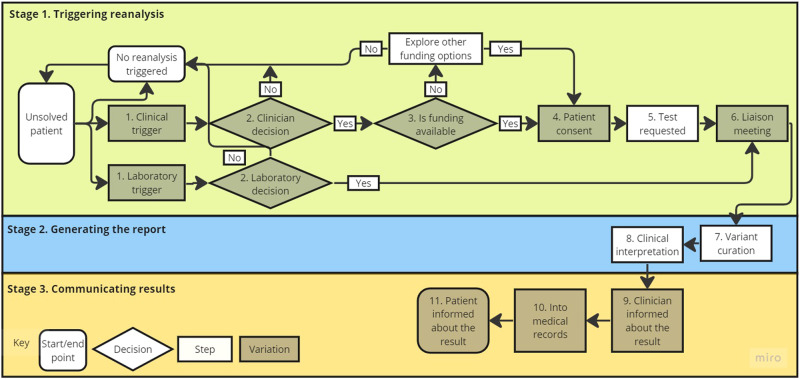
Summarised current manual approach to reanalysis process map. Stage 1 of the process includes 'triggering reanalysis' starting with an unsolved patient, stage 2 indicates the steps to 'generating the report' and stage 3 shows the steps to 'communicating the results' ending with the patient informed about the results. Key: the **start and end point** of the process are symbolised by a rounded edge box, **steps** are right angled boxes, **decision points** are diamonds and **darkended shapes** indicate variation.

Individual process maps were produced for each interview using standardised symbols (See Fig. 1 legend). Team member (ZF) assessed with ongoing meetings with (SB) the individual process maps and used *Miro* (Miro.com) to generate a manual and automated summary map. Team members (ZF, SB, ZS) met to review, edit, and approve the final outputs. To identify perceived challenges, transcripts were analysed deductively using the CFIR coding guide [13]. Following familiarisation with transcripts, sections of text describing each step were organised accordingly. Once in the framework, data within each step was then coded to the CFIR independently by two team members (ZF, SB) who met to discuss and resolve discrepancies in coding. Next, CFIR codes were matched with the top three ranking ERIC strategies using the available tool [14]. Finally, experience-based examples of how the strategy could be enacted were drawn from the interview data.

## Results

### Characteristics of participants

Of the 19 participants identified, 14 were interviewed (Table 1). Two individuals did not respond, one felt they lacked patient experience with reanalysis and two became unavailable. One interview was conducted with two participants (one clinical and one laboratory) working at the same service.

**Table 1:** Participant characteristics.

Participant characteristic, *n* (%)	
Role	
Clinical Geneticists (CG)	5 (36%)
Genetic Counsellor (GC)	5 (36%)
Clinical Scientist (Lab)	4 (28%)
Patient cohort	
Paediatric	7 (50%)
Adult	2 (14%)
Both	5 (36%)
Area	
Rare disease	12 (86%)
Cancer	2 (14%)

### Current manual process and variation

Figure 1 presents the summarised map. Three stages of reanalysis (1: Triggering Reanalysis; 2: Generating the Report; and 3: Communicating Results) were identified through analysis of the thirteen process maps (see Supplementary Material 2). In stage 1, three patient pathways associated with initiating manual reanalysis were discussed. On top, and the pathway most frequently taken, was that no reanalysis for a patient was triggered. Underneath, a clinical trigger whereby reanalysis is initiated in a clinical setting was reported as the predominant reanalysis pathway and included eleven steps. On the bottom and the least frequent pathway was a laboratory trigger, whereby a laboratory initiates the possible reanalysis and included nine steps.

Variation was noted within eight steps (grey tiles) and are reported in Table 2. For example, *Step 2 Clinician Decision* some clinics had eligibility guidelines, whereas others used clinician discretion and *Step 3 Funding for Reanalysis* varied between state government funding, hospital, clinic, or laboratory service budget, or through research projects. *Step 4 Patient Consent* could either be obtained formally or informally at a review appointment and at some cancer services was included in the original consent form.

**Table 2:** Variation in the steps for the current manual process.

Step	Variation
Step 1	Clinician books in or advises their patient to return in 2–3 years
	At patient/family request
	Another specialist involved in a patient’s care initiates request or rerefers patient to genetic service
	Clinician request shortly after primary test (prenatal setting)
	Laboratory flags cases where reanalysis may yield a diagnosis
Step 2	Clinic or laboratory guideline
	Clinician discretion
	Consulting clinicians’ discretion
	Laboratory discretion (laboratory workforce capacity, volume of patients that would benefit)
Step 3	Clinic or Laboratory department budget (may require other clinicians’ approval)
	State Government funded
	Research projects
Step 4	Formal or informal reconsent
	Part of the original consent form (cancer)
Step 6	Liaison meetings between clinical teams and laboratories (in *some* departments)
Step 9	New or amended report
	Email, fax, phone
	If laboratory triggered it might be an informal communication
Step 10	Electronically entered in ERM
	Manually entered in electronic or paper medical record
Step 11	Automatically booked into a return of results appointment
	Patient/family offered appointment to discuss finding
	No new findings via telephone call

### Proposed automated process

**Fig. 2:**
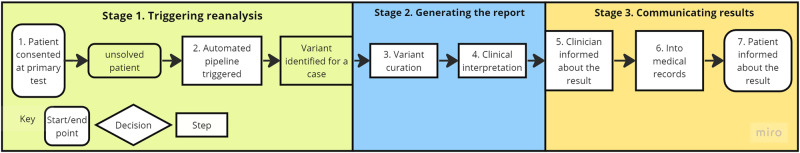
Summarised automated approach to reanalysis process map. Stage 1 of the process includes 'triggering reanalysis' starting with patient consented at primary test, stage 2 indicates the steps to 'generating the report' and stage 3 shows the steps to 'communicating the results' ending with the patient informed about the results. Key: the **start and end point** of the process are symbolised by a rounded edge box, **steps** are right angled boxes, and **decision points** are diamonds.

Repeating process mapping for a proposed automated approach shows a streamlined process with one patient pathway and the number of steps reduced to seven (Fig. 2). From stage 1, clinical and laboratory decision-making steps were removed, and funding becomes part of the programme design. Overall, participants supported automated reanalysis and discussed the patient and family benefits, and many reflected that automation ultimately reduces workloads as one participant put it,

“I don’t think anyone has the workforce in the lab or in the clinical genetics’ services…whereas if we automate the process and patients could be consented once and the pipeline runs, suddenly the existing workforce could do this piece of work in addition to newly requested tests, and it would pick-up findings that would otherwise sit in the dark”. Lab07

### Identifying possible implementation strategies to overcome perceived challenges

Although welcoming, challenges exist at six of the steps. To identify potential implementation strategies, we present challenges organised according to the step in the automate process, coded to the CFIR and **associated ‘ERIC strategy’ (bolded in text)**, with an experienced-based example of the strategy drawn from the interview data (Table 3). Exemplar quotes are presented in text using identifiers (CG, GC, and Lab) and in Supplementary Material 3.

**Table 3:** Challenges organised by step in the automated reanalysis process, coded to the CFIR, matched to top rated ERIC strategies and experience-based example of the strategy.

Step	Challenges	CFIR code	Top rated ERIC strategies	Experience-based example
**Step 1** Patient consented for reanalysis at primary test	**Unknown consent process**. How to opt out, reconsent at age of transition, or analysis scope is broadened, and when offered in mainstream services	Design, Quality & Packaging	Promote adaptability	**Clinical team education** *…clarity around the lab processes and good education to the different clinical units about the process so they can then interpret that in a way that they can educate their patients*. GC14
			Develop educational materials	
			Obtain & use patient/consumer/family feedback	
**Step 2** Automated pipeline triggered	**Updating clinical information**. No mechanism to update the pipeline with new clinical information	Available Resources	Access new funding	**A clinical information feed platform and teamworking with primary care physicians** *Unless, you have some amazing platform that allows physicians and health care providers who have ongoing contact with the patient and family to be able to add in additional information*. GC18
			Change physical structure & equipment	
			Fund & contract for clinical innovation	
	**Trust in the automated pipeline**. That the pipeline is triggered and performs as expected	Knowledge & Beliefs	Conduct educational meetings	**Local champions at services** *…it won’t be until it is implemented then people will see the benefit and that instils trust…I think having champions at each site that are really engaged with the project and can help almost guide those clinicians*. GC14
			Identify & prepare champions	
			Develop educational materials	
		Executing	Purposely re-examine the implementation	**An audit process** *From our [the laboratory] point of view how do we sufficiently engage the software to know that the right things are being completed and then making a log of that process*. Lab10
			Develop & implement tools for quality monitoring	
			Provide local technical assistance	
**Step 3** Variant curation	**Unknown laboratory workforce implications and skills shortage**.	Executing	Purposely re-examine the implementation	**Develop and test the pipeline** *It comes down to having the right tool that allows us [the laboratory] a fairly hands free or even eyes free way of knowing what we need to follow-up*. Lab11
			Develop & implement tools for quality monitoring	
			Provide local technical assistance	
**Step 4** Clinical interpretation	**Clinical workforce capacity**. Lack of funding towards attending Multi-Disciplinary Team (MDTs) meetings	Available Resources	Access new funding	**Additional renumeration** *We already are involved in attending MDTs but our role is going to evolve … especially as patients are now going to be having reanalysis through our services, as long as that is funded and acknowledged*. CG05
**Step 5** Clinician informed about the result	**Ensuring the report is received by a clinician who can action it**.	Compatibility	Promote adaptability	**Reporting pathway** *The lab would need to have an agreement with each department…where there was a monitored inbox where reanalysed reports go so that if someone moves on or is on maternity leave for a year that report doesn’t get lost in the ether*. GC06
			Conduct local consensus discussions	
			Conduct cyclical small tests of change	
**Step 6** Into medical records	*None reported*			
**Step 7** Patient informed about the result	**Clinical workforce implications**. Managing expectations, locating patients/families and, results return appointments	Individual Stage of Change	Identify and prepare champions	**Staffing levels and workforce infrastructure** *You wouldn’t always need clinical geneticist to be involved in the return of results to families, skilled genetic counsellors can be involved in that process. So thinking about how to best utilise the workforce*. CG03
			Make training dynamic	
			Alter incentive/allowance structures	
	**Processes for recontacting patients/ families**.	Executing	Purposely re-examine the implementation	**A national approach** *If there was sone kind of national approach or database where we could track patients down easier* GC17
			Develop & implement tools for quality monitoring	
			Provide local technical assistance	
**Funded reanalysis programme**	**Lack of an appropriate funding model**.	External policies & incentives	Involve executive boards	**Appropriate funding model** *You would need a completely new funding model. It needs to work at scale, so the more samples you process, each individual one becomes cheaper to get each answer*. CG01
			Alter incentive/allowance structures	
			Build a coalition	

#### Step 1: Patient consented at primary test

There was agreement amongst participants that consent for reanalysis would become incorporated into pre-test counselling. Required programme design elements currently presenting as a challenge (CFIR: Design, Quality, & Packaging) were establishing an opt-out function “*we will have to have that capacity to turn it off”* (Lab10) and the opportunity for patients to provide consent around the time they transition to adult care, or if the scope of analysis was broadened. Supporting non-genetic health care professionals to deliver adequate pre-test counselling was raised by some participants. Experience-based examples supporting clarity around processes and education matched with ERIC strategy **‘Develop Educational Materials’**. For the scenarios requiring variation, **‘Promoting Adaptability’** may aid implementation. Further, **‘Obtaining Consumer Feedback’** could enhance consent processes.

#### Step 2: Automated pipeline triggered

Triggering reanalysis without a mechanism to update clinical information was a perceived barrier (CFIR: Available Resources). Establishing a clinical information platform and teamworking with health care professionals who have ongoing care was intuitively discussed and links with ERIC strategies **‘Fund and Contract for Clinical Innovation’**. By removing the clinician-initiated request, gaining trust that pipeline performs as intended was emphasised (CFIR: Knowledge & Beliefs About the Intervention). **‘Develop Education Materials’** and **‘Identify and Prepare Local Champions’** are possible implementation strategies. Likewise, an audit process (CFIR: Executing) was proposed to build trust and links with ERIC strategies to **‘Examine Implementation’** and **‘Develop Quality Monitoring Tools’**.

#### Step 3: Variant curation

Whilst variant curation would follow existing laboratory guidelines, the unknown implications of enacting an automated pipeline on laboratory workforces were discussed and some sites reported skills shortages (CFIR: Executing). **‘Purposively Re-examining the Implementation’,** **‘Develop and Implement Tools for Quality Monitoring’**, and **‘Provide Local Technical Assistance’** are potential implementation strategies.

#### Step 4: Clinical interpretation

An automated pipeline would likely increase clinical staff attendance at laboratory multi-disciplinary team meetings which currently lacks a funding avenue (CFIR: Available Resources). **‘Access new funding’** aside, no intuitive example was provided.

#### Step 5: Clinician informed about the results

With a once-off test request, ensuring that a new actionable finding reached either the requesting clinician or someone who could action it was a perceived barrier by clinical teams and laboratory staff (CFIR: Compatibility). **‘Promoting Adaptability’** or **‘Conducting Local Consensus Discussion’** between laboratory and clinical teams and testing the new workflow in **‘Small Change Cycles’** may facilitate the change in test request pathways.

#### Step 6: Into medical records

*None reported*.

#### Step 7: Patient informed about the result

With the clinician-initiated request removed, some clinical staff felt an automated approach would increase workloads by needing to manage ongoing patient expectations, factoring in return of results appointments and the time spent locating patients. On the other hand, some clinicians felt by reducing review appointments which are currently required to trigger manual requests for reanalysis they would have more availability (CFIR: Individual Stage of Change). **‘Altering Allowances Structures’** or increasing clinical workforce funding is a possible avenue or as one participant identified better utilising the current workforce. Likewise, embedding project **‘Champions’** or **‘Making Training Dynamic’** may overcome workforce hesitancy. Issues around locating patients and families to return results and the extent of the legal and ethical obligation were discussed by clinical staff (CFIR: Executing). Once underway, **‘Purposively Re-examining the programme Implementation’,** **‘Developing Quality Monitoring Tools’** and **‘Providing Assistance’** are potential strategies to ensure the step can be achieved. Outside of the ERIC strategies, several participants promoted the idea of a national database that could be accessed for recontacting purposes.

#### Funded reanalysis programme

Whilst no longer a step, funding was a prominent barrier (CFIR: External Policies & Incentives). Participants discussed the lack of an appropriate funding model and the pitfalls of the current fee-for-service model. Aside from **‘Altering Allowances Structures’** working alongside **‘Executive Boards’** or **‘Building a Coalition’** are linked ERIC strategies that may promote the development of a suitable funding mechanism.

## Discussion

The results of this study demonstrate that automating reanalysis has the potential to streamline processes, reduce variation and optimise resource use. Using two theoretical frameworks, our study moves beyond reporting challenges to automating reanalysis raised by participants and identifies implementation strategies that target barriers and may enhance future adoption, implementation, and sustainability [23]. Additionally, several of the theory-informed implementation strategies aligned with experience-based examples raised by participants. Being exploratory in nature, we intend for this study to provide theory-informed implementation science guidance for future efforts and contribute to the evidence supporting implementation of genomic medicine [24, 25].

Participants reported that reanalysis is infrequently performed and through process mapping we were able to articulate *why* services are unable to meet need. The current ‘manual’ approach relies on a highly motivated clinician or patient and family to attend a review appointment. Once seen, clinical decision-making points and differences in funding models increases the number of steps required to access reanalysis. Likewise, laboratories undertake several steps reviewing requests prior to reanalysing a case. Complexity, including duration, intricacy, and number of steps, is a known cause of unintended consequences and factors into implementation failure [26, 27]. Importantly, automated reanalysis was shown to remove decision-making steps, reducing the complexity and clinical and laboratory staff’s cognitive load. Overall, clinical and laboratory staff were welcoming of an automated approach to reanalysis, however switching from a manual, clinician-requested approach to an automated pipeline disrupts existing work routines and raised reservations.

Adapting to new ways of working is not without challenges and understanding stakeholders’ perception towards the ‘implementability’ of an innovation is recognised to influence uptake [28]. Challenges were raised by participants during interviews whilst discussing an automated workflow “*so thinking about it in that linear process, one barrier will be…*” (GC06) indicating process mapping is a useful method to ascertain some of the complexities in the process. Clinical and laboratory staff perceived challenges at all but one of the process steps. From our analysis, CFIR coded challenges represent each of the five domains (Innovation Characteristics, Outer Setting, Inner Setting, Characteristics of Individuals, and Process). This finding aligns with previous research that demonstrates the implementation of genomic medicine requires a coordinated effort that tackles numerous aspects at once [29]. Whilst only a handful of the CFIR constructs were identified (seven out of a possible 37), they are in keeping with barriers reported in the literature e.g., workforce capacity, [7, 8, 10] reimbursement structures, [3] consent and recontacting patients, [4–6] bioinformatics and information technology systems, [4] and the implementation of genomic medicine more broadly [30]. We extend this work by applying two theoretical frameworks (CFIR and ERIC) to categorise challenges and identify targeted implementation strategies.

Establishing an appropriate funding mechanism was a prominent barrier that we coded to CFIR ‘External Policies and Incentives’ as participants spoke of a funding mechanism within a public health system. We acknowledge that different settings will require individual consideration towards the implications of automated reanalysis on reimbursement structures and relevant strategies. A recently updated version of the CFIR (CFIR_2) [31] separates ‘External Polices and Incentives’ into several constructs including ‘Financing’ and may provide the granularity required across settings. ‘Executing’ was a recurrent CFIR factor, which may not be surprising during effectiveness-implementation hybrid type studies whereby an innovation is tested alongside the gathering information on the implementation context and the potential for future use [18]. As promoted by the ERIC strategies, structured assessment of the performance of automated reanalysis pipelines and ongoing quality monitoring may help build clinical and laboratory staff trust, confidence, and positive perceptions towards the innovation [32]. Creating a supportive social environment through embedding project champions can also help overcome initial workforce hesitancy and target individual level challenges. Champions can fulfil various roles and responsibilities (e.g., front-line, or managerial) that will be service context specific [27]. Whilst the step ‘Into Medical Records’ (Fig. 2) did not present as a barrier to participants, in other genomic programmes it is a priority area [33].

Utilising frameworks proved advantageous by providing a nuanced understanding of perceived challenges and insight on ‘how to’ go about enabling change [23]. Drawing on theory explains the reason why the implementation strategy may produce the desired outcome [34]. For example, clinical and laboratory workforce implications were both perceived challenges, yet they differed in CFIR coding. Clinical workforce barriers were categorised to ‘Individual Stage of Change’ indicating the types and level of engagement and educational strategies required. Laboratory workforce capacity related to ‘Executing’, suggesting encouraging a small cycles approach to trialling automated reanalysis would be beneficial. This difference suggests which implementation strategies will be required and as previously described, [35] shows the value of a step-wise approach rather than broadly applying strategies across situations.

Although theoretical frameworks such as the ERIC are useful tools to guide and select strategies, one critique of this approach is the insufficient description on how strategies can be operationalised [36]. One solution is to involve end-users’ in the design and refinement. Rather than actively seeking their expertise which can be time consuming, [37] our results show that clinical and laboratory staff intuitively suggested possible examples during interviews. For the most part the experience-based examples conceptually linked with theory and so can be capitalised on to direct next steps. For example, to support the adoption of a new consent process (CFIR: Design Quality & Packaging) the ‘Development of education materials’ was both a matched ERIC strategy and intuitively suggested in interviews. Participants also raised the importance of education for clinical departments following the establishment of laboratory processes. However, ‘Obtain & Use Patient/Consumer/Family Feedback’ whilst a related ERIC strategy and is beneficial to developing ‘consumer‐informed’ materials, [38] it was not discussed in interviews. Another example of how integrating theory and experience may be beneficial is that whilst we opted to report the three top ranking ERIC strategies for each barrier, using the tool in this way may not have yielded the best selection. Again, involving end-users upfront in the selection process can be leveraged and lead to selections with consideration of contextual factors and preferences [39, 40].

The study limitations include the small sample of Australian services within a public healthcare system we drew upon. However, process mapping can be reproduced in other contexts, as can matching the ERIC strategies to CFIR constructs, for example [33]. We elected to use the original CFIR framework to exploit the related ERIC matching tool. However, the updated CFIR_2 may provide opportunity to expand on some of our inferences. Finally, we did not capture the consumer experience and perspective nor the view of other health care professionals who offer genomic testing. Future work to ascertain the perspective of these groups is underway.

We report clinical and laboratory staff’s firsthand experience of delivering reanalysis in services across Australia. Our findings show why a manual approach to reanalysis is unsustainable and how an automation can improve service delivery. To realise the benefits of automation, we have identified how implementation strategies that target challenges, are contextually relevant, and align with end-user preferences may be a useful addition to implementation efforts.

### Supplementary information

Supplementary Material 1

Supplementary Material 2

Supplementary Material 3

## Data Availability

The datasets generated during and/or analysed during the current study can be made available from the corresponding author upon reasonable request.
