# Supplementary material for: Reanalysis of genomic data, how do we do it now and what if we automate it? A qualitative study
Source: Eur J Hum Genet. 2024 Jan 12;32(5):521–8. doi: 10.1038/s41431-023-01532-4 (PMC11061153; doi:10.1038/s41431-023-01532-4)
Supplement: Supplementary file 1 — Supplementary Material 1 [file 41431_2023_1532_MOESM1_ESM.docx]

Supplementary Material 1: Interview schedule

| I would like to begin by concentrating on the current manual approach to reanalysis using the process map I shared previously and will now share with you. (Start screen sharing the process map) |
| --- |
| **Unsolved patient** |
| How does the reanalysis process get started?- what is the trigger?  What proportion of your patients have data reanalysed?  Which patients have their data reanalysed? – all? How often? Till when?  How do you decide who to reanalyse? Are there any clinical criteria? e.g., monogenic, reproductive plans of parents etc.,? |
| Has a routine process been established for reanalysis? Do you have to make a formal lab request?  Or is there an informal process?  How do you manage this? |
| How are patients consented (and whose role it it)? Do patients have to be reconsented or can you use the original consent form? |
| **Reanalysis** |
| How is reanalysis currently funded? (How do you think automated reanalysis should be funded) |
| If you get a positive result? How does a result get to clinicians and into the medical record |
| What happens if there is a negative result?  Are they automatically reanalysed? If so, how often? |
| **Patient informed about results** |
| How do you inform the patients about their result? (positive or negative) |
| Have there been (are you aware of) any issues around communicating reanalysis results back to patients? |
| *No*: What has been put in place to ensure it worked well?  *Yes*: What has been challenging? What needs to be put in place to overcome this? |
| What are your thoughts for it being automated? *stop sharing and w*ork through the process map again* |
| **Process** |
| What do you think will be the challenges to implementing automated reanalysis as part of routine practice? |
| What do you feel should be put in place (if anything) ensure **automated** reanalysis happens?  How could this be facilitated? |
| **Patient** |
| What do patients say to you about reanalysis of their data? Expressed expectations? |
| What impact do you think automating reanalysis will have on patients? |
| **Personal** |
| Are there any implications (for your patients or department) for not automating reanalysis? |
| What sort of role do you envisage you will need to play with automated reanalysis? |
| How do you feel about the role you will have to play in the reanalysis process? |
| **Organisational** |
| What is the feeling in your lab/dept about automating reanalysis |
| Is your organisation supportive of automating reanalyse of genomic data |
| Are there any other challenges, maybe one you have overcome, that we haven’t discussed and you would like to share? |
