# Supplementary material for: Reanalysis of genomic data, how do we do it now and what if we automate it? A qualitative study
Source: Eur J Hum Genet. 2024 Jan 12;32(5):521–8. doi: 10.1038/s41431-023-01532-4 (PMC11061153; doi:10.1038/s41431-023-01532-4)
Supplement: Supplementary file 2 — Supplementary Material 2 [file 41431_2023_1532_MOESM2_ESM.pptx]

## Slide 1
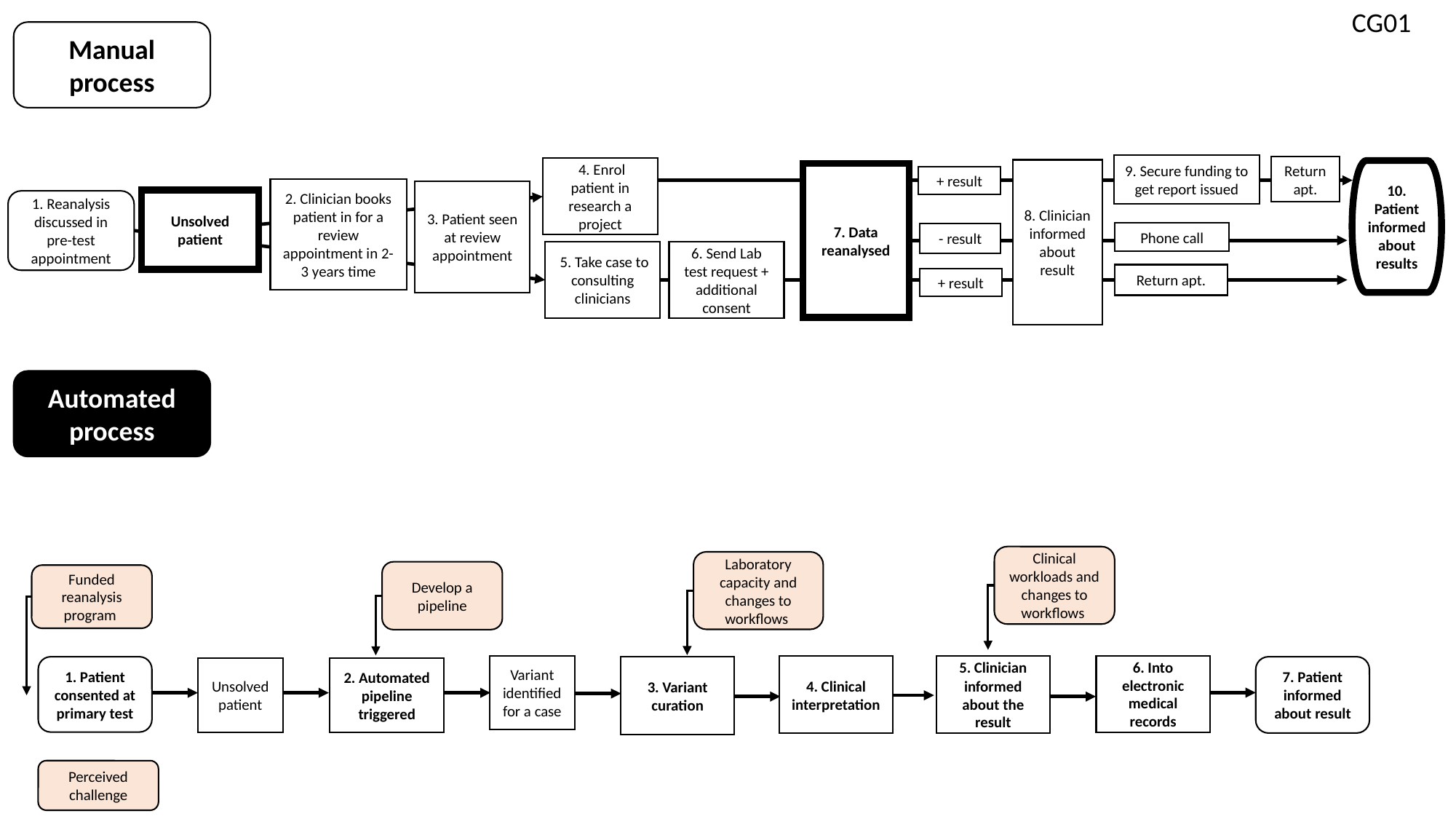

CG01
Manual process
9. Secure funding to get report issued
Return apt.
 4. Enrol patient in research a project
8. Clinician informed about result
10. Patient informed about results
7. Data reanalysed
+ result
2. Clinician books patient in for a review appointment in 2-3 years time
3. Patient seen at review appointment
Unsolved patient
1. Reanalysis discussed in pre-test appointment
Phone call
- result
6. Send Lab test request + additional consent
 5. Take case to consulting clinicians
Return apt.
+ result
Automated process
Clinical workloads and changes to workflows
Laboratory capacity and changes to workflows
Develop a pipeline
Funded reanalysis program
Variant identified for a case
4. Clinical interpretation
5. Clinician informed about the result
6. Into electronic medical records
7. Patient informed about result
1. Patient consented at primary test
3. Variant curation
Unsolved patient
2. Automated pipeline triggered
Perceived challenge

## Slide 2
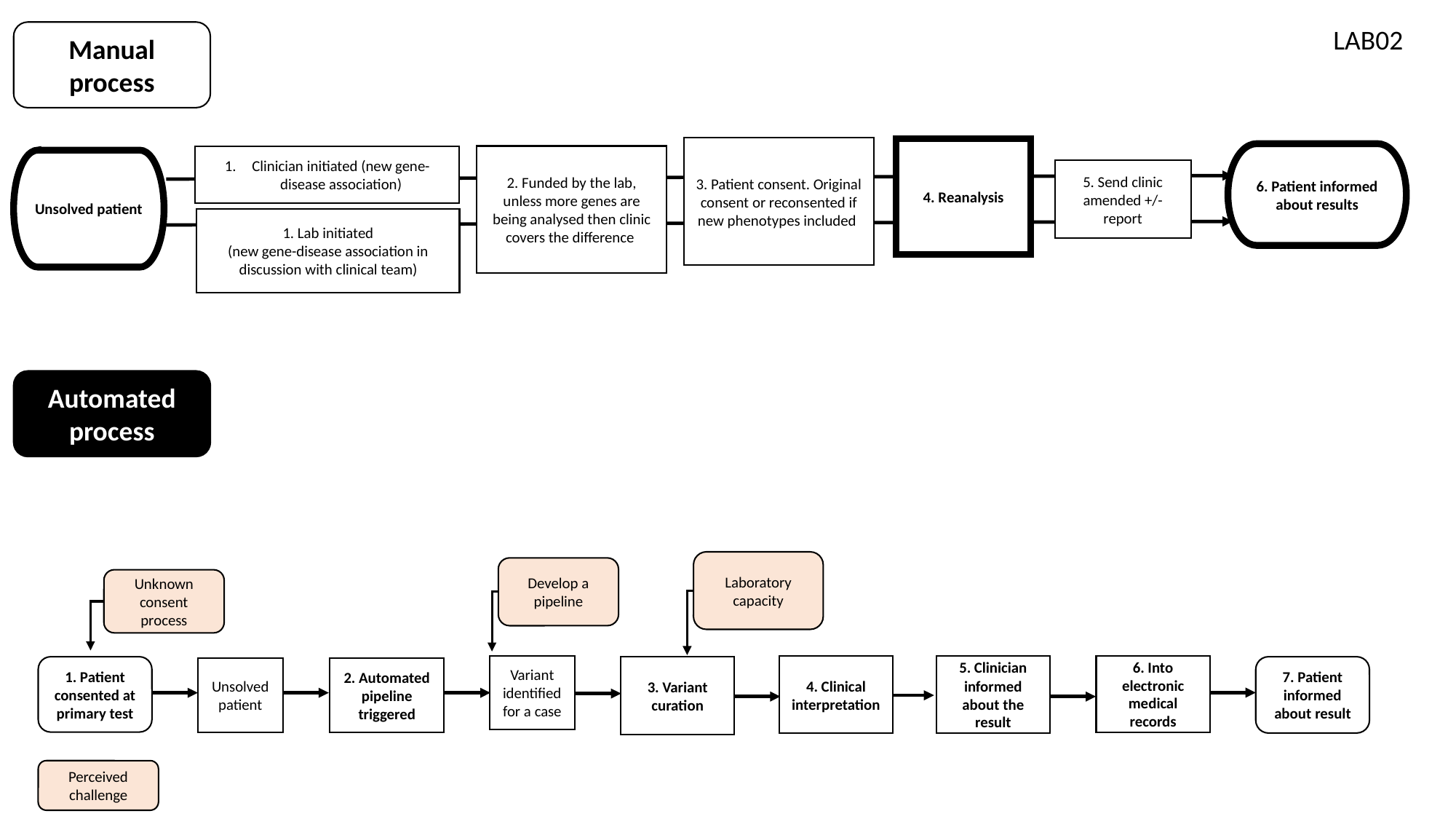

LAB02
Manual process
3. Patient consent. Original consent or reconsented if new phenotypes included
4. Reanalysis
6. Patient informed about results
Unsolved patient
Clinician initiated (new gene-disease association)
2. Funded by the lab, unless more genes are being analysed then clinic covers the difference
5. Send clinic amended +/- report
1. Lab initiated
(new gene-disease association in discussion with clinical team)
Automated process
Laboratory capacity
Develop a pipeline
Unknown consent process
Variant identified for a case
4. Clinical interpretation
5. Clinician informed about the result
6. Into electronic medical records
7. Patient informed about result
1. Patient consented at primary test
3. Variant curation
Unsolved patient
2. Automated pipeline triggered
Perceived challenge

## Slide 3
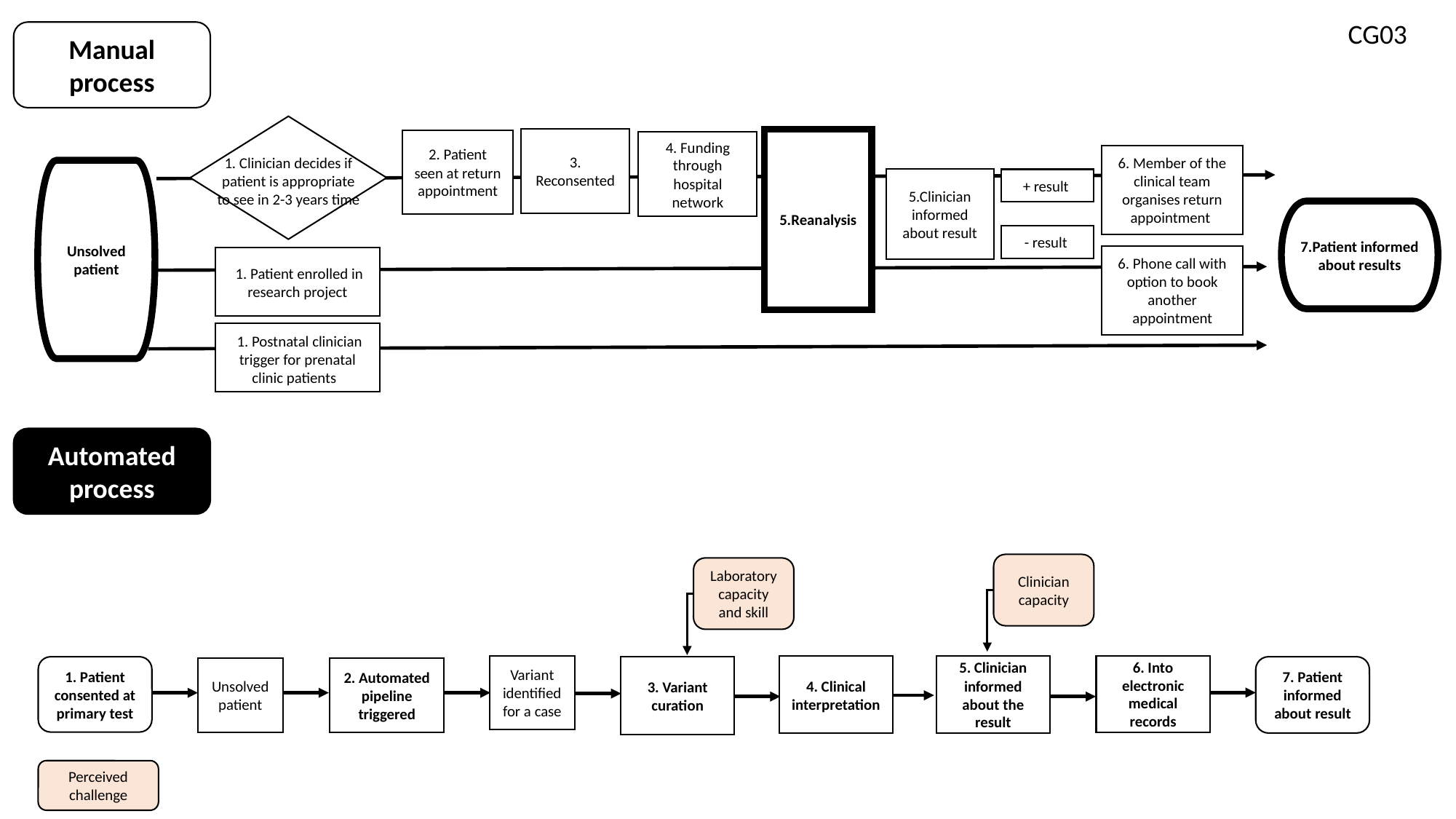

CG03
Manual process
1. Clinician decides if patient is appropriate to see in 2-3 years time
3. Reconsented
5.Reanalysis
2. Patient seen at return appointment
4. Funding through hospital network
6. Member of the clinical team organises return appointment
Unsolved patient
5.Clinician informed about result
+ result
7.Patient informed about results
- result
6. Phone call with option to book another appointment
 1. Patient enrolled in research project
 1. Postnatal clinician trigger for prenatal clinic patients
Automated process
Clinician capacity
Laboratory capacity and skill
Variant identified for a case
4. Clinical interpretation
5. Clinician informed about the result
6. Into electronic medical records
7. Patient informed about result
1. Patient consented at primary test
3. Variant curation
Unsolved patient
2. Automated pipeline triggered
Perceived challenge

## Slide 4
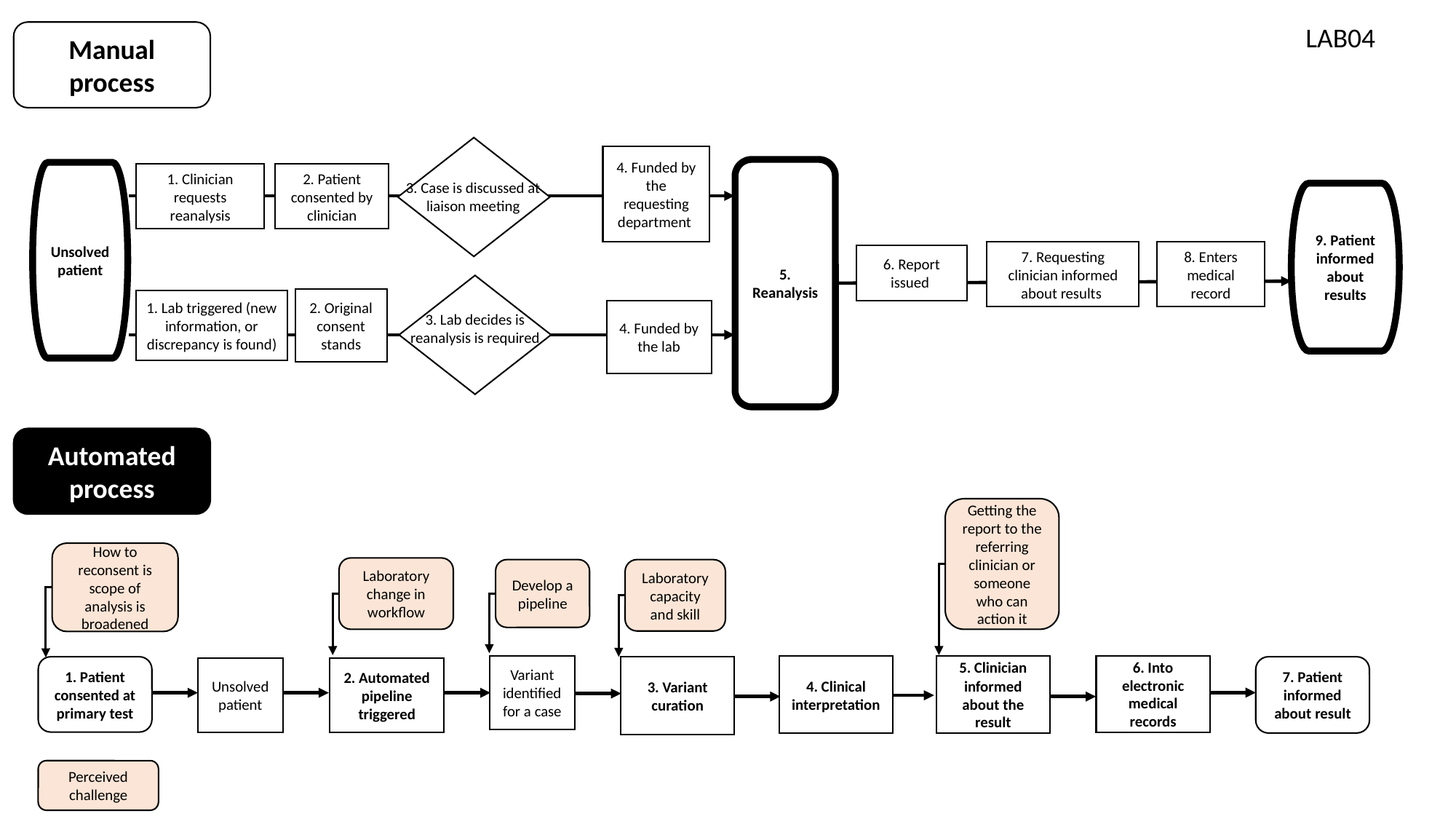

LAB04
Manual process
3. Case is discussed at liaison meeting
4. Funded by the requesting department
5. Reanalysis
Unsolved patient
9. Patient informed about results
1. Clinician requests reanalysis
2. Patient consented by clinician
7. Requesting clinician informed about results
8. Enters medical record
6. Report issued
2. Original consent stands
1. Lab triggered (new information, or discrepancy is found)
4. Funded by the lab
3. Lab decides is reanalysis is required
Automated process
Getting the report to the referring clinician or someone who can action it
How to reconsent is scope of analysis is broadened
Laboratory change in workflow
Develop a pipeline
Laboratory capacity and skill
Variant identified for a case
4. Clinical interpretation
5. Clinician informed about the result
6. Into electronic medical records
7. Patient informed about result
1. Patient consented at primary test
3. Variant curation
Unsolved patient
2. Automated pipeline triggered
Perceived challenge

## Slide 5
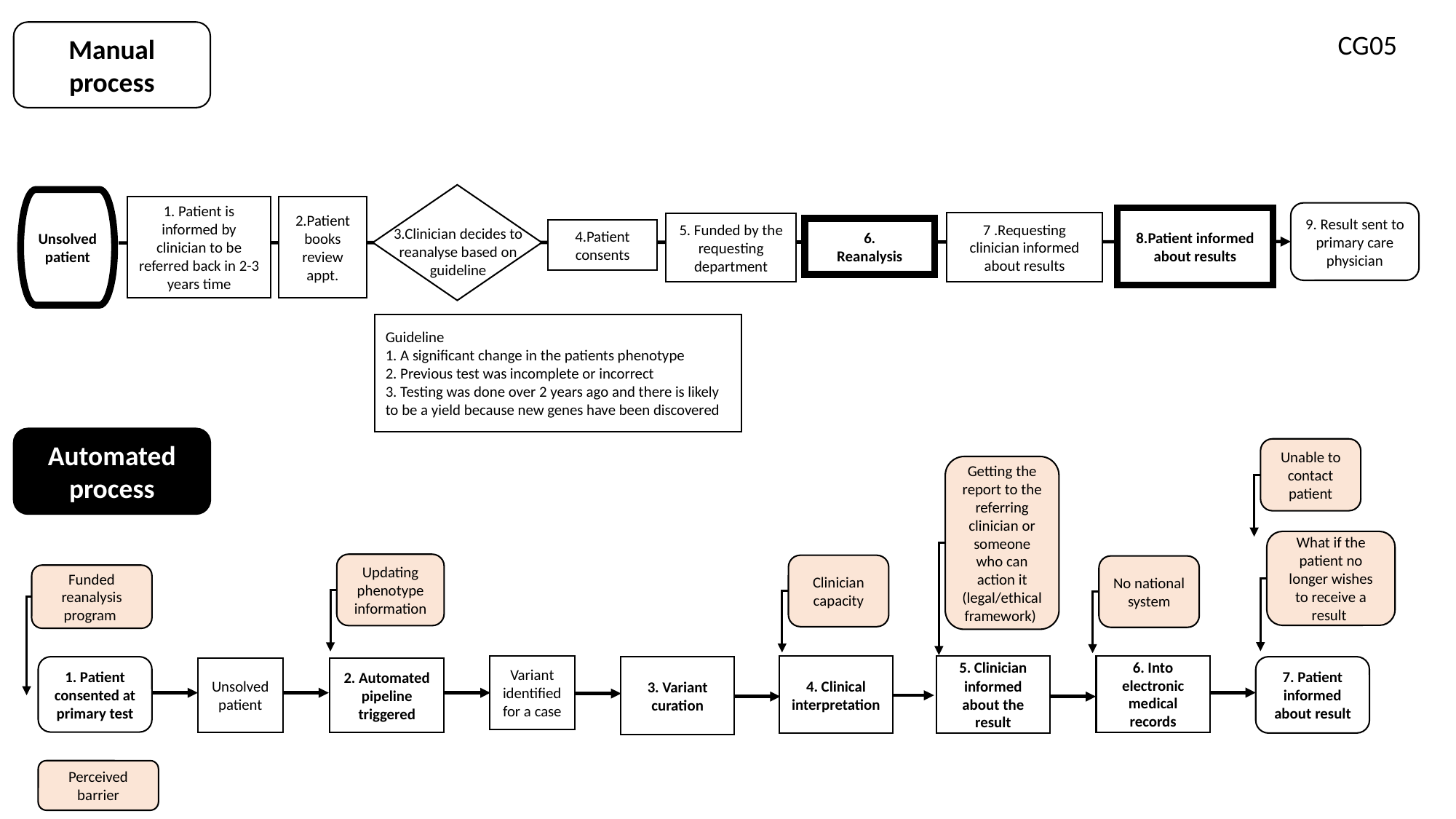

Manual process
CG05
3.Clinician decides to reanalyse based on guideline
Unsolved patient
8.Patient informed about results
6.
Reanalysis
2.Patient books review appt.
1. Patient is informed by clinician to be referred back in 2-3 years time
9. Result sent to primary care physician
7 .Requesting clinician informed about results
5. Funded by the requesting department
4.Patient consents
Guideline
1. A significant change in the patients phenotype
2. Previous test was incomplete or incorrect
3. Testing was done over 2 years ago and there is likely to be a yield because new genes have been discovered
Automated process
Unable to contact patient
Getting the report to the referring clinician or someone who can action it (legal/ethical framework)
What if the patient no longer wishes to receive a result
Updating phenotype information
Clinician capacity
No national system
Funded reanalysis program
Variant identified for a case
4. Clinical interpretation
5. Clinician informed about the result
6. Into electronic medical records
7. Patient informed about result
1. Patient consented at primary test
3. Variant curation
Unsolved patient
2. Automated pipeline triggered
Perceived barrier

## Slide 6
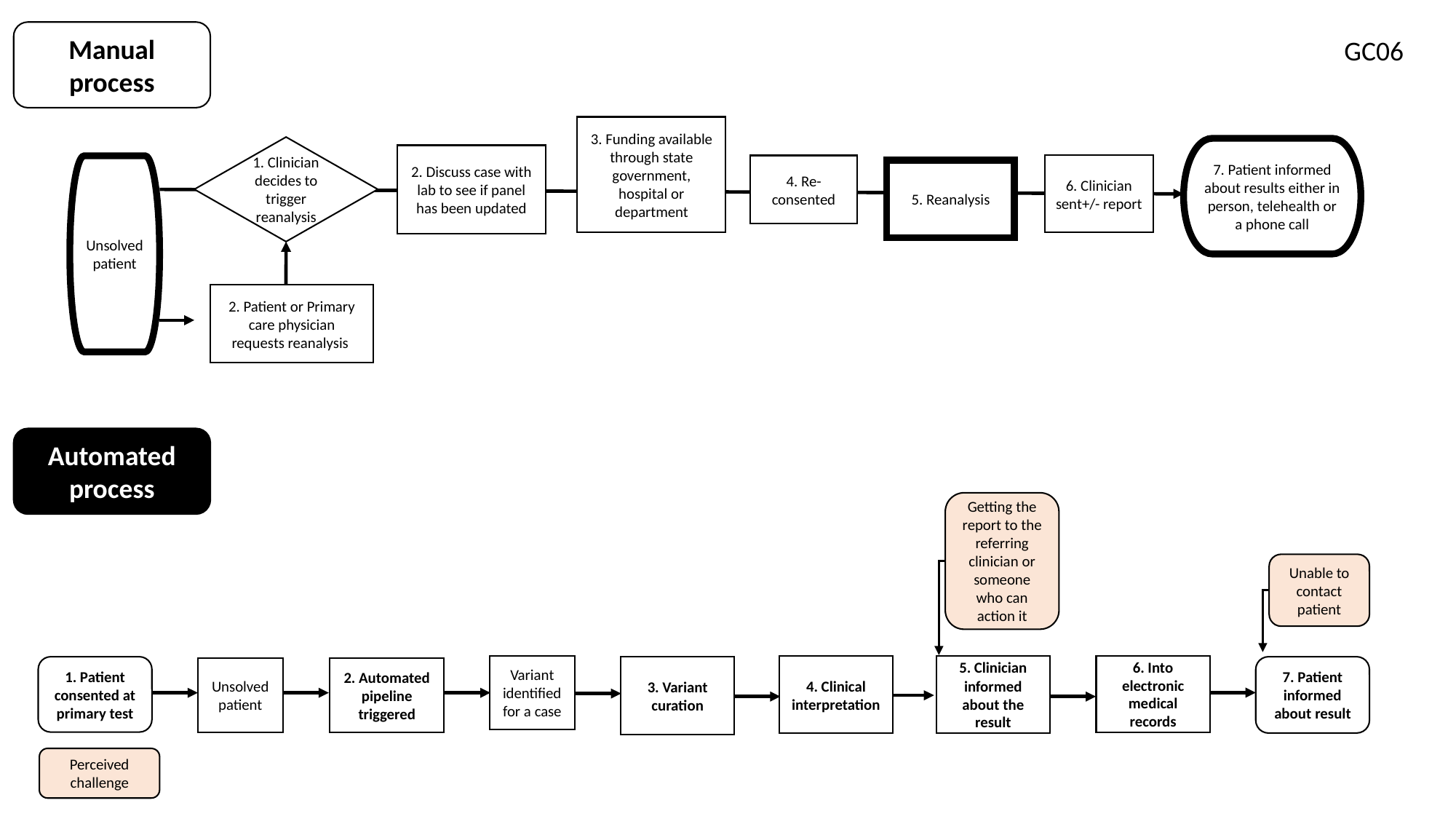

Manual process
GC06
3. Funding available through state government, hospital or department
1. Clinician decides to trigger reanalysis
7. Patient informed about results either in person, telehealth or a phone call
Unsolved patient
5. Reanalysis
2. Discuss case with lab to see if panel has been updated
4. Re-consented
6. Clinician sent+/- report
2. Patient or Primary care physician requests reanalysis
Automated process
Getting the report to the referring clinician or someone who can action it
Unable to contact patient
Variant identified for a case
4. Clinical interpretation
5. Clinician informed about the result
6. Into electronic medical records
7. Patient informed about result
1. Patient consented at primary test
3. Variant curation
Unsolved patient
2. Automated pipeline triggered
Perceived challenge

## Slide 7
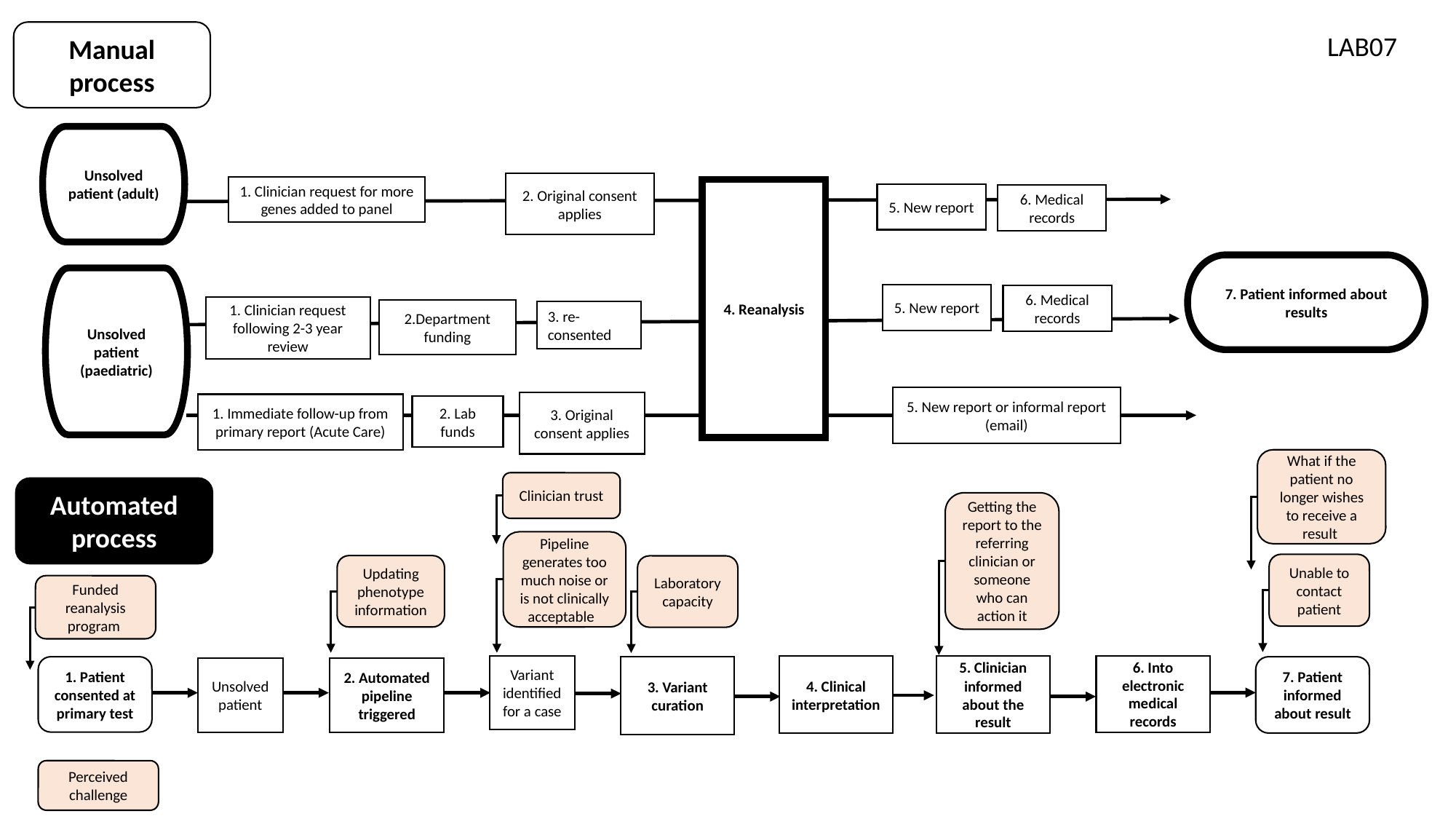

Manual process
LAB07
Unsolved patient (adult)
2. Original consent applies
1. Clinician request for more genes added to panel
4. Reanalysis
5. New report
6. Medical records
7. Patient informed about results
Unsolved patient (paediatric)
5. New report
6. Medical records
1. Clinician request following 2-3 year review
2.Department funding
3. re-consented
5. New report or informal report (email)
3. Original consent applies
1. Immediate follow-up from primary report (Acute Care)
2. Lab funds
What if the patient no longer wishes to receive a result
Clinician trust
Automated process
Getting the report to the referring clinician or someone who can action it
Pipeline generates too much noise or is not clinically acceptable
Unable to contact patient
Updating phenotype information
Laboratory capacity
Funded reanalysis program
Variant identified for a case
4. Clinical interpretation
5. Clinician informed about the result
6. Into electronic medical records
7. Patient informed about result
1. Patient consented at primary test
3. Variant curation
Unsolved patient
2. Automated pipeline triggered
Perceived challenge

## Slide 8
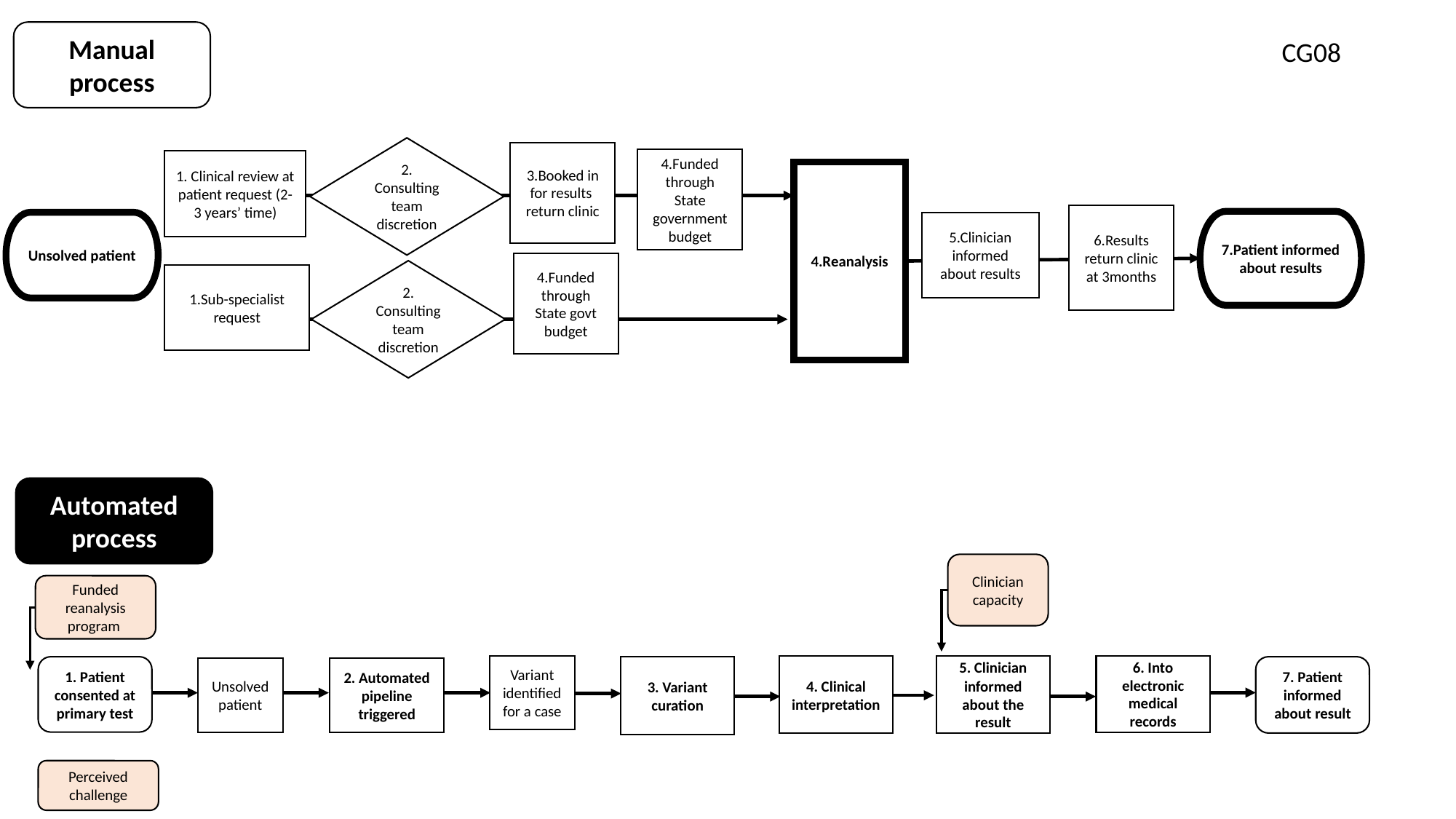

Manual process
CG08
2. Consulting team discretion
3.Booked in for results return clinic
4.Funded through State government budget
1. Clinical review at patient request (2-3 years’ time)
4.Reanalysis
7.Patient informed about results
Unsolved patient
6.Results return clinic at 3months
5.Clinician informed about results
4.Funded through State govt budget
2. Consulting team discretion
1.Sub-specialist request
Automated process
Clinician capacity
Funded reanalysis program
Variant identified for a case
4. Clinical interpretation
5. Clinician informed about the result
6. Into electronic medical records
7. Patient informed about result
1. Patient consented at primary test
3. Variant curation
Unsolved patient
2. Automated pipeline triggered
Perceived challenge

## Slide 9
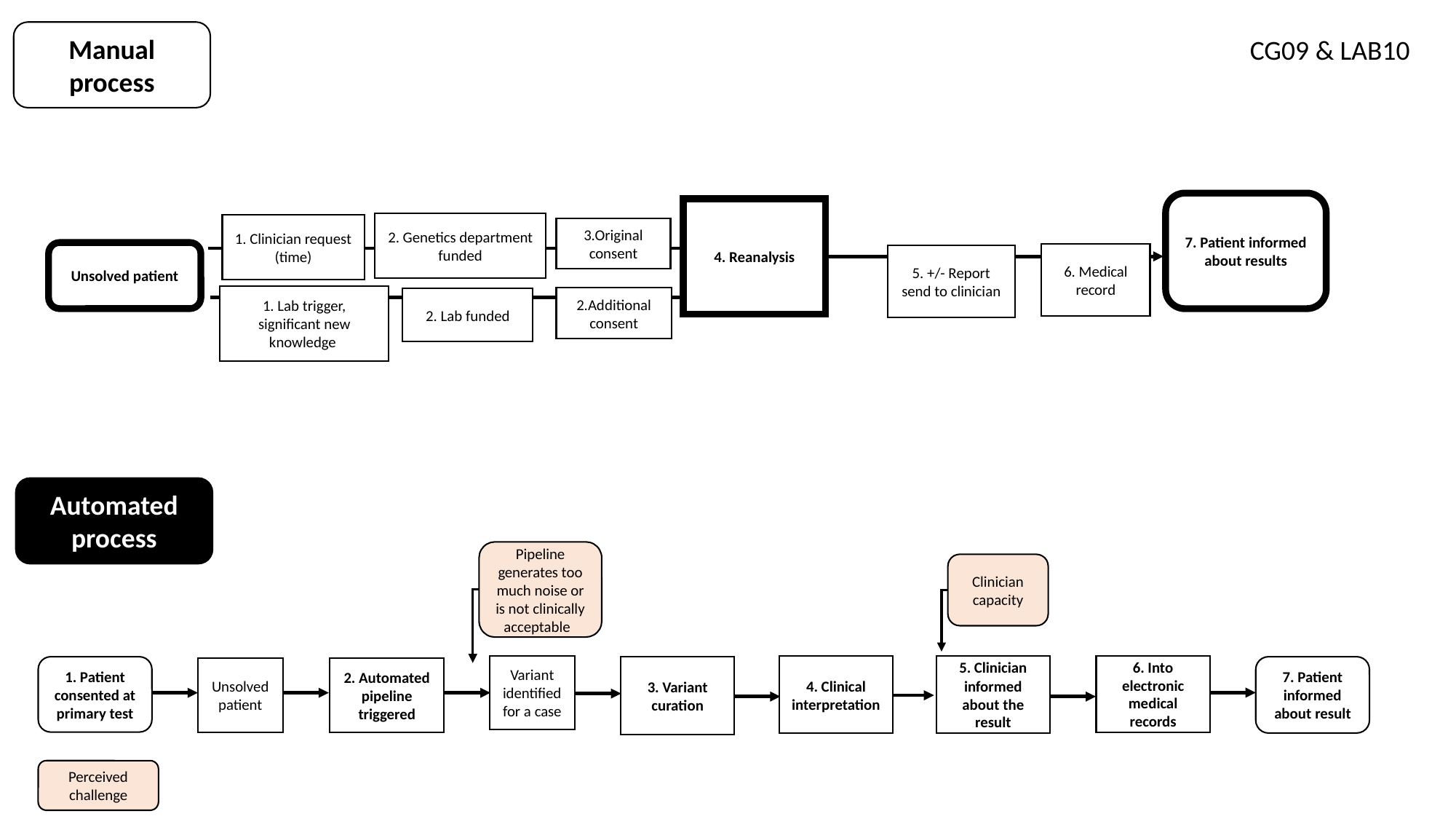

Manual process
CG09 & LAB10
7. Patient informed about results
4. Reanalysis
Unsolved patient
2. Genetics department funded
1. Clinician request (time)
3.Original consent
6. Medical record
5. +/- Report send to clinician
1. Lab trigger, significant new knowledge
2.Additional consent
2. Lab funded
Automated process
Pipeline generates too much noise or is not clinically acceptable
Clinician capacity
Variant identified for a case
4. Clinical interpretation
5. Clinician informed about the result
6. Into electronic medical records
7. Patient informed about result
1. Patient consented at primary test
3. Variant curation
Unsolved patient
2. Automated pipeline triggered
Perceived challenge

## Slide 10
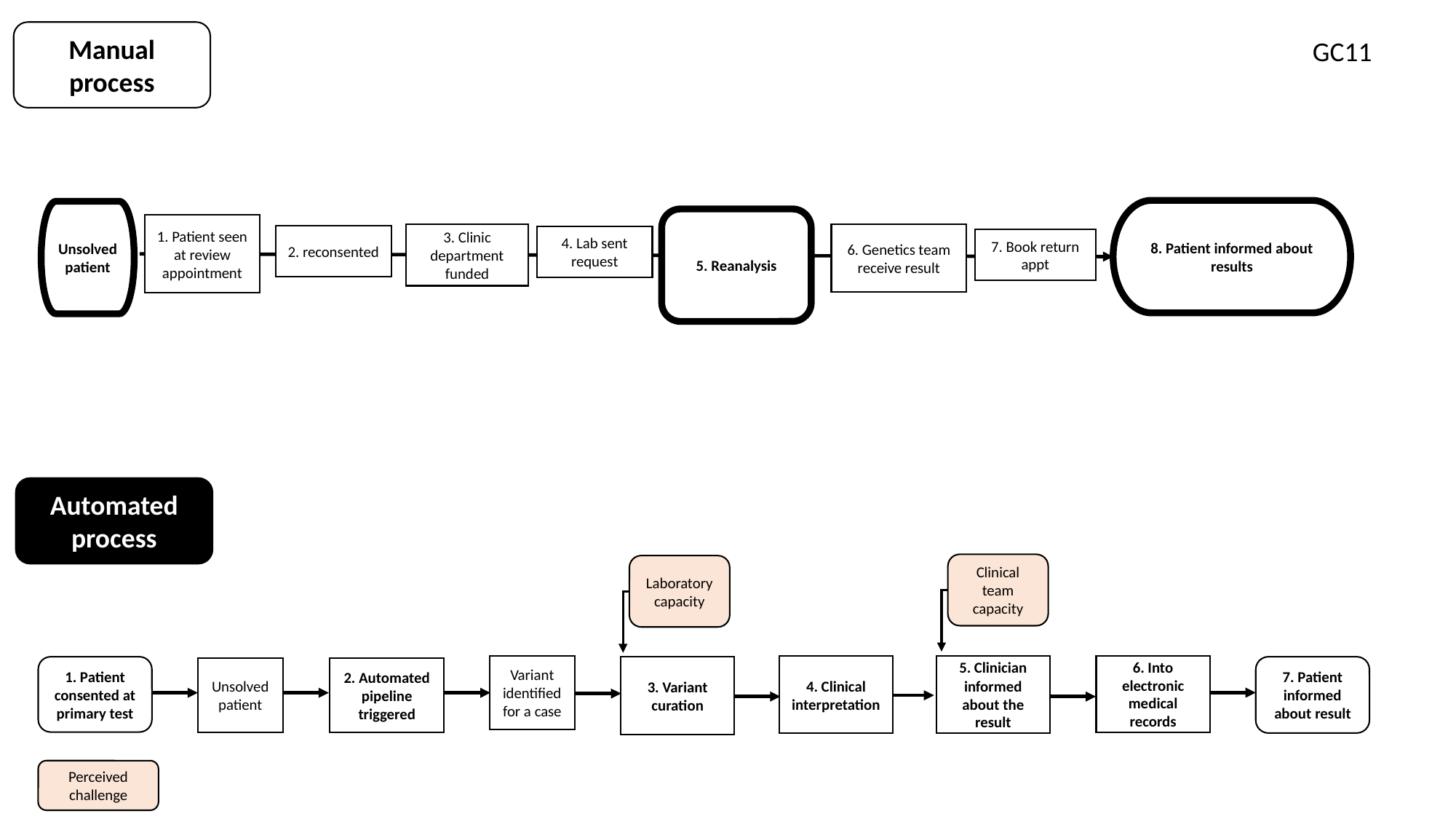

Manual process
GC11
8. Patient informed about results
Unsolved patient
5. Reanalysis
1. Patient seen at review appointment
3. Clinic department funded
6. Genetics team receive result
2. reconsented
4. Lab sent request
7. Book return appt
Automated process
Clinical team capacity
Laboratory capacity
Variant identified for a case
4. Clinical interpretation
5. Clinician informed about the result
6. Into electronic medical records
7. Patient informed about result
1. Patient consented at primary test
3. Variant curation
Unsolved patient
2. Automated pipeline triggered
Perceived challenge

## Slide 11
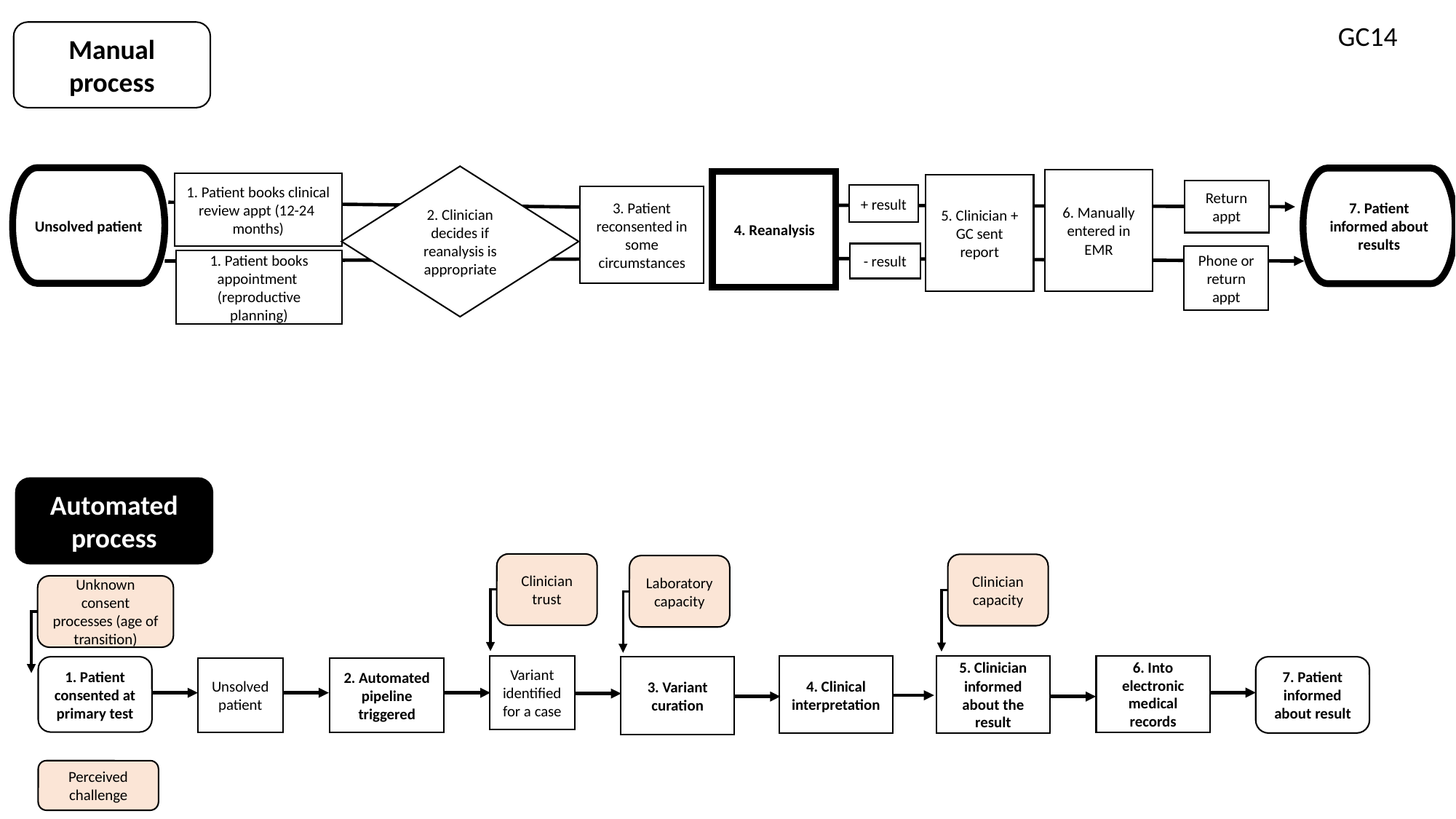

GC14
Manual process
2. Clinician decides if reanalysis is appropriate
Unsolved patient
7. Patient informed about results
4. Reanalysis
6. Manually entered in EMR
1. Patient books clinical review appt (12-24 months)
5. Clinician + GC sent report
Return appt
+ result
3. Patient reconsented in some circumstances
- result
Phone or return appt
1. Patient books appointment
(reproductive planning)
Automated process
Clinician trust
Clinician capacity
Laboratory capacity
Unknown consent processes (age of transition)
Variant identified for a case
4. Clinical interpretation
5. Clinician informed about the result
6. Into electronic medical records
7. Patient informed about result
1. Patient consented at primary test
3. Variant curation
Unsolved patient
2. Automated pipeline triggered
Perceived challenge

## Slide 12
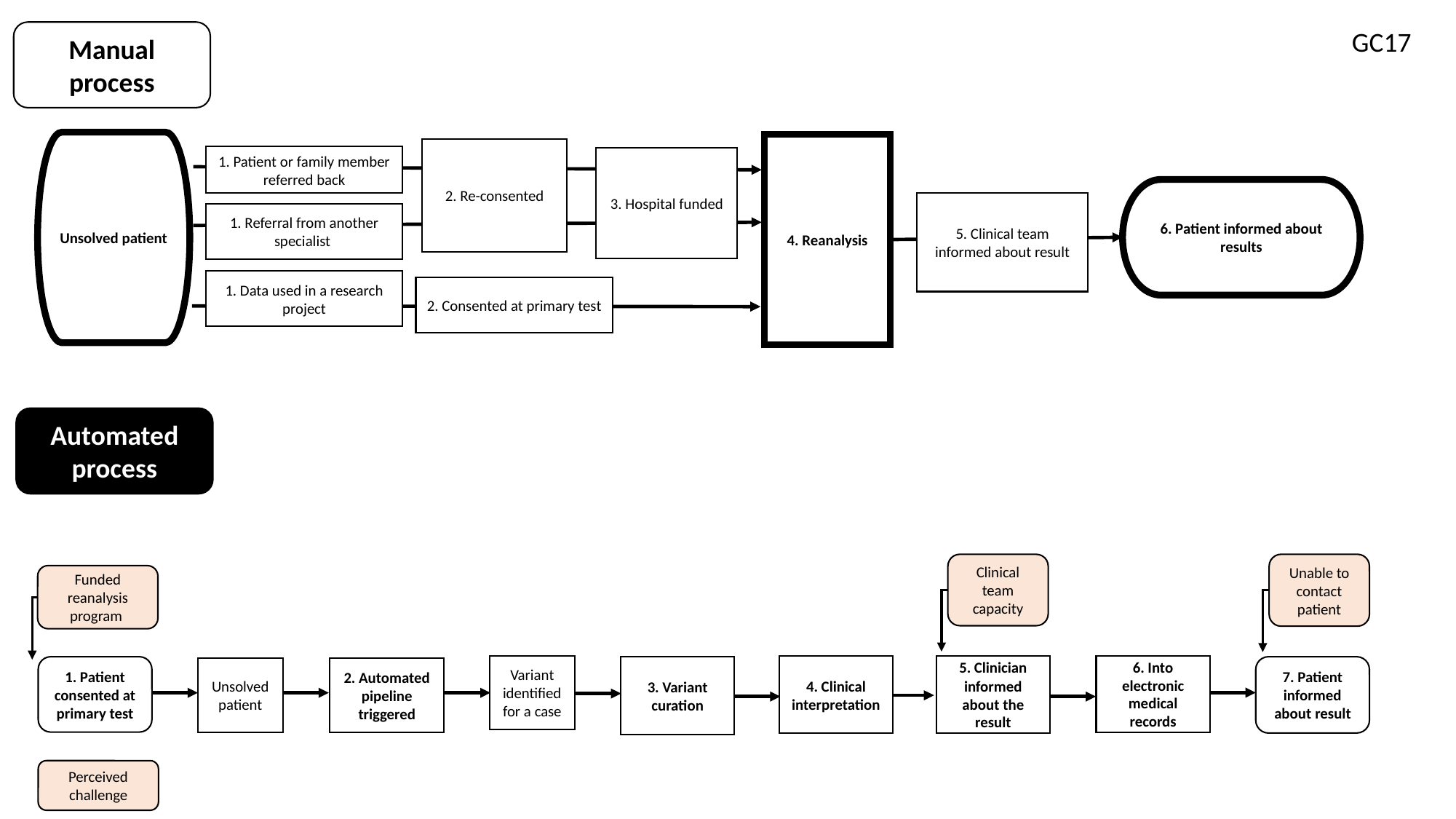

GC17
Manual process
Unsolved patient
4. Reanalysis
6. Patient informed about results
2. Re-consented
1. Patient or family member referred back
3. Hospital funded
5. Clinical team informed about result
1. Referral from another specialist
1. Data used in a research project
2. Consented at primary test
Automated process
Clinical team capacity
Unable to contact patient
Funded reanalysis program
Variant identified for a case
4. Clinical interpretation
5. Clinician informed about the result
6. Into electronic medical records
7. Patient informed about result
1. Patient consented at primary test
3. Variant curation
Unsolved patient
2. Automated pipeline triggered
Perceived challenge

## Slide 13
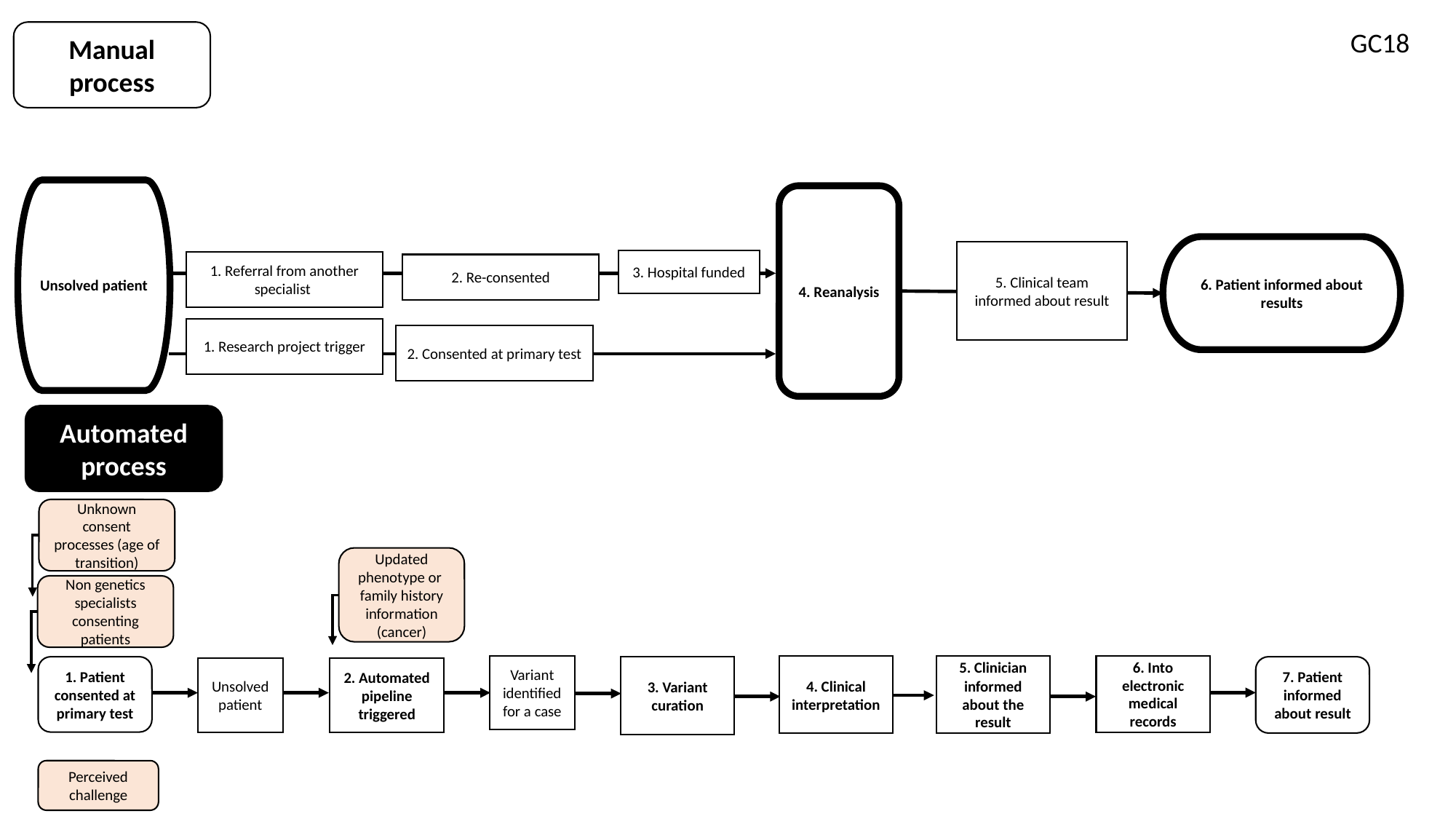

GC18
Manual process
Unsolved patient
4. Reanalysis
6. Patient informed about results
5. Clinical team informed about result
3. Hospital funded
1. Referral from another specialist
2. Re-consented
1. Research project trigger
2. Consented at primary test
Automated process
Unknown consent processes (age of transition)
Updated phenotype or family history information (cancer)
Non genetics specialists consenting patients
Variant identified for a case
4. Clinical interpretation
5. Clinician informed about the result
6. Into electronic medical records
7. Patient informed about result
1. Patient consented at primary test
3. Variant curation
Unsolved patient
2. Automated pipeline triggered
Perceived challenge
