# Supplementary material for: Reanalysis of genomic data, how do we do it now and what if we automate it? A qualitative study
Source: Eur J Hum Genet. 2024 Jan 12;32(5):521–8. doi: 10.1038/s41431-023-01532-4 (PMC11061153; doi:10.1038/s41431-023-01532-4)
Supplement: Supplementary file 3 — Supplementary Material 3 [file 41431_2023_1532_MOESM3_ESM.docx]

Supplementary Material 3: Challenges coded to CFIR with definition by process step and exemplar quotes.

| **Step** | **Challenge** | **CFIR code** and definition | **Exemplar quotes** |
| --- | --- | --- | --- |
| **Step 1:** Patient consented at primary test | **Unknown consent process.** Including how to opt out, reconsent at age of transition, or if scope of analysis is broadened, and when offered by non-genetics professionals | **Design, Quality & Packaging.** The degree to which the innovation is well designed and packaged, including how it is assembled, bundled, and presented | One concern I had was around consent for the patient, how can we be sure the patient wants auto reanalysis? Lab07 |
|  |  |  | Some families may not want that future contact, so it might be important to have that option for opting out. Although they might opt out, but it might have important implications for other family members. But that is still their choice. CG03 |
|  |  |  | I think we will have to have that capacity to turn it off and a way of recognising someone who wants to know vs those - we’re past having our children. Lab10 |
|  |  |  | I wonder how that works if you haven’t had the opportunity to reconsent them once they have become an adult…that is an issue that would need to be resolved for an automated pipeline. GC18 |
|  |  |  | How will this [automated reanalysis] work outside of a genetics clinic where you have got non-genetic health professionals who have ordered testing and having reanalysis. That is tricky because I would worry that, that discussion hadn’t been made clear to a family without a genetic counsellor present. GC14 |
| **Step 2:** Automated pipeline triggered | **Updating clinical information.** No mechanism to update the pipeline with new clinical information | **Available Resources.** The extent to which resources (e.g., funding, space, materials and equipment) are sufficient to support the implementation of the innovation. | I think automated reanalysis will have its limitations and I don’t think it will allow for any automated update of phenotype change. GC18 |
|  |  |  | Updated clinical information to allow for the automated reanalysis to be effective and increase that diagnostic yield...We need to sort out how we are going to get updated clinical information… we all have different medical records in each state, some are electronic, some aren’t. So, the lab can’t even just pull that [clinical] information from electronic records automatically. CG05 |
|  | **Trust in the automated pipeline.** | **Knowledge & Beliefs About the Intervention.** The extent to which stakeholders have positive attitudes, they place high value on, and/or they are familiar with facts, truths, and principles about the innovation | …you have to get clinicians to trust in the system. Which I think is a pretty big hurdle for clinicians who are used to do things one way and then bring in all this new stuff, it can get pretty overwhelming. GC14 |
|  |  |  | I think ensuring the clinical validity of the results…if you miss the clinically significant variant then there is no point in having it done because you are almost luring the doctor into a sense of negative testing…it will be hard for the clinician to know whether to trust the auto analysis pathway. Lab07 |
|  |  | **Executing.** Implementation activities are/ are not being done according to plan | We would need to audit what has been reanalysed. So yep, we had 50 patients, yep that’s all go through and some sort of audit check…[to] know it’s performing according to its set characteristics. Lab10 |
| **Step 3:** Variant curation | **Unknown laboratory workforce implications and skills shortage.** | **Executing.** (See above) | That will be one of the main sticking points, having the time to look at results from the automated reanalysis. Lab02 |
|  |  |  | It [the pipeline] couldn’t generate a lot of noise…it’s got to work for the workforce that is there. So, if its constantly throwing up and stuff and you’re going – ahh Stop! Stop! That would be really bad. CG09 |
|  |  |  | There is a little bit of skills shortage in variant curation at the moment, so we need new people and there isn’t a lot of education out there for the new people although it is improving. Lab04 |
| **Step 4:** Clinical interpretation | **Clinical workforce capacity.** Lack of funding towards attending Multi-Disciplinary Team (MDTs) meetings | **Available Resources.** (See above) | ...if [the laboratory] suddenly comes out and says - I’ve got 10 new variants for 10 of your patients, let’s do a MDT. I have to factor that into my normal workflow, and I don’t have funding for that additional bit of work. CG05 |
| **Step 5:** Clinician informed about the result | **Ensuring the report is received by a clinician who can action it** | **Compatibility.** The extent to which the innovation fits well with existing workflows or with the meaning and values attached to the innovation, or with stakeholders’ own needs and/or it reduces/ heightens the risk for stakeholders. | One of the barriers will be ensuring the report goes to a clinician and is seen by someone who will action it. GC06 |
|  |  |  | If it’s an automated process and the labs comes back with a result, who is it going to go to? Is it going to go to the clinician who requested the test, that might be genetics or non-genetics and what about if they are no longer looking after the patient? Is it then the labs legal responsibility to chase that clinician? Like that result will be lost in the ether, it will go as an attachment in an email. Here is your updated report. What if the patient never finds out? Where is the ethical, legal framework here? CG05 |
| **Step 6:** Into medical records | *None reported* |  |  |
| **Step 7: Patient** informed about the result | **Processes for recontacting patients/families.** | **Executing.** (See above) | The next barrier is contacting the family, finding the family, and if we are talking about months, years down the track then of course people move house, change phone numbers, emails and that will be a difficult one. GC06 |
|  |  |  | When it comes to informing the patient, what if the patient has moved interstate? Or I can’t get hold of them. How am I going to tell them? That legal responsibility…What do I do with the result? CG05 |
|  | **Clinical workforce implications.** Managing expectations, locating patients/families and, results return appointments | **Individual stage of change.** Characterization of the phase an individual is in, as he or she progresses toward skilled, enthusiastic, and sustained use of the intervention | My fear is, that we as genetic counsellors because we are very easy to contact from the patients and the families, is that they will be constantly calling us saying - is there anything yet? Is there anything yet? And that can take a lot of time when we are already time poor and resource poor. GC14 |
|  |  |  | I would say people are nervous and that is because I think…people are so overwhelmed with their current work because of the resourcing issues of not having enough genetic counsellors. Not having enough clinical geneticist. GC14 |
|  |  |  | I think the concept is very sound from the diagnostic perspective but I think the management of it needs a lot of work. And the predominant thing is being able to offer follow-up that is required around these results. CG08 |
|  |  |  | I think people are worried about getting results and that increasing their workload and I sit back and look at that and think – how can we possible be worried about delivering the one thing that our patients and families want. CG01 |
| **Funding model in place** | **Lack of an appropriate funding model** | **External policies & incentives.** The extent to which external policies, regulations (governmental or other central entity), mandates, recommendations or guidelines, pay-for-performance, collaborative, or public or benchmark reporting exist, or they help efforts to implement the innovation. | The funding model would be a big challenge because it can’t be spread on a patient-by-patient basis. CG01 |
|  |  |  | We all want to do well for our patients, but we also work in an infrastructure where we have funding constraints, activity constraints. So, who is going to pay for this? Unless this is nationally funded, but I can’t see that happening anytime soon. CG05 |
